# The Association between Neutrophil-to-Lymphocyte Ratio and Glycemic Control in Type 2 Diabetes Mellitus: A Systematic Review and Meta-Analysis

**DOI:** 10.1155/2023/3117396

**Published:** 2023-06-03

**Authors:** Tiruneh Adane, Mulugeta Melku, Yilkal Belete Worku, Alebachew Fasil, Melak Aynalem, Amanuel Kelem, Solomon Getawa

**Affiliations:** ^1^Department of Hematology and Immunohematology, School of Biomedical and Laboratory Sciences, College of Medicine and Health Sciences, University of Gondar, Gondar, Ethiopia; ^2^Department of Internal Medicine, School of Medicine, College of Medicine and Health Sciences, University of Gondar, Gondar, Ethiopia; ^3^Department of Clinical Chemistry, School of Biomedical and Laboratory Sciences, College of Medicine and Health Sciences, University of Gondar, Gondar, Ethiopia; ^4^Department of Medical Laboratory Sciences, Asrat Woldeyes Health Science Campus, Debre Berhan University, Debre Berhan, Ethiopia

## Abstract

**Background:**

Glycated hemoglobin (HbA1c) is a commonly used clinical marker to monitor the control of type 2 diabetes mellitus patients (T2DM). However, it is unable to identify the ongoing inflammatory changes in the body. These factors could be easily identified and monitored by the neutrophil-to-lymphocyte ratio (NLR). Therefore, this study is aimed at investigating the relationship between NLR and glycemic control in T2DM.

**Method:**

A comprehensive search of eligible studies was performed in various databases published until July 2021. A random effect model was used to estimate the standardized mean difference (SMD). A metaregression, subgroup, and sensitivity analysis were conducted to search for potential sources of heterogeneity.

**Result:**

A total of 13 studies were included in this study. Accordingly, the SMD of the NLR values between the poor and good glycemic control groups was 0.79 (95% CI, 0.46-1.12). Our study also showed that high NLR was significantly associated with poor glycemic control in T2DM patients (OR = 1.50, 95% CI: 1.30-1.93).

**Conclusion:**

The results of this study suggest an association between high NLR values and an elevated HbA1C in T2DM patients. Therefore, NLR should be considered a marker of glycemic control in addition to HbA1c in T2DM patients.

## 1. Introduction

Diabetes mellitus (DM) is a multifaceted metabolic disorder that affects the body's blood glucose levels [1]. Based on insulin dependency, it can be classified into type 1 DM (T1DM) and T2DM [2]. T2DM is an inflammatory disease with immune system dysfunction [3]. Low-grade inflammation plays a significant role in the pathogenesis of T2DM, particularly in the development of insulin resistance associated with obesity [4]. Chronic inflammation, indicated by an elevated leukocyte count, may play a central role in the development of diabetic macro- and microvascular complications [5]. Type 2 DM is also associated with changes in serum levels of inflammatory markers like mean platelet volume (MPV) [6] and cytokines [7]. It is well known that T2DM patients are recommended to maintain glycemic standards based on epidemiological data to prevent, or at least delay, the onset and progression of vascular complications [8].

The HbA1c measures average glycemia over about three months and aids in determining the disease's level of management [9]. Poorer outcomes during the course of the disease are associated with higher HbA1c levels [10]. One of the most often utilized tests to check on the management of DM is the HbA1c [11]. It is unable to identify the ongoing inflammatory changes in the body, though. The neutrophil-to-lymphocyte ratio (NLR) can easily identify and monitor such conditions [12].

The NLR is a reliable biomarker of low-grade inflammation in various clinical conditions such as hypertension, metabolic syndrome, obesity, and lifestyle changes [13]. Elevated NLR has also been reported in various inflammatory diseases including type 2 diabetes mellitus [14], irritable bowel disease [15], cancer [16], inflammatory bowel disease [17], cardiac conditions [18], thyroiditis [19], and COVID-19 infection [20]. NLR has emerged as a novel indicator of systemic inflammatory response in various diseases in recent years. In many clinical settings, NLR is considered an independent predictor of major morbidity, mortality, and long-term survival [21]. Besides, it can also be used for population screening, disease detection, and drug monitoring [22]. Neutrophils are the main branch of leukocytes in the bloodstream. Initially, they respond rapidly to the inflammatory stimuli, and the neutrophil count increases in circulation. Instead, interleukin levels that increase in inflammatory conditions cause lymphopenia and neutrophilia, together causing elevated NLR [23, 24].

NLR represents neutrophil and lymphocyte; the 2 components of chronic inflammatory condition [25]. A high neutrophil value is a marker of the ongoing, destructive, nonspecific inflammatory process. Conversely, a low lymphocyte count indicates relatively inadequate immune regulation as well as a quiescent immunity pathway [26]. Hence, a high level of NLR can indicate the functional status of the immune system in the course of chronic inflammation [27]. However, NLR is relatively more stable and less influenced by physiological, pathological, and physical factors than individual leukocyte parameters [28]. The NLR is a low cost, widely available parameter that has been investigated as a reliable proxy marker of systemic inflammation in a spectrum of chronic diseases [29, 30].

Recently, the relationship between DM and NLR has also become a current issue of investigation [31]. Therefore, the main aim of this systematic review and meta-analysis is to investigate the potential role of NLR as an indicator of glycemic control in T2DM patients.

## 2. Method

### 2.1. Design and Protocol Registration

This systematic review and meta-analysis were conducted as per the 2020 PRISMA guidelines [32]. The protocol has been registered in the International Prospective Register of Systematic Reviews (PROSPERO), with the registration number CRD42021273819.

### 2.2. Eligibility Criteria

Articles were included in the meta-analysis if they met each of the following criteria: (1) cross-sectional, case-control, and cohort studies published in peer-reviewed journals evaluating the relationship between NLR and glycemic control in T2DM patients; (2) full text in English; (3) published online up to July 2021; and (4) expressing NLR results as mean and standard deviation (SD) and/or median and interquartile range (IQR). We have excluded studies with (1) insufficient or ambiguous data for meta-analysis; (2) overlapping or duplicate data; and (3) poster presentations, reviews, case reports, and editorial letters.

### 2.3. Search Strategy

We conducted a comprehensive search of eligible studies in the PubMed/MEDLINE, Cochrane Library, Google Scholar, Scopus, Web of Science, and EMBASE published until July 2021. It was strengthened by searching the reference lists of published articles to identify relevant unpublished studies. The search strategy was based on the combinations of keywords and medical subject heading (MeSH) terms as follows: “neutrophil lymphocyte ratio” or “NLR” or “neutrophil-to-lymphocyte ratio” AND “glycemic control” or “glucose regulation” “level of HgA1C” AND “DM” or “diabetes mellitus” or “Type 2 diabetes.”

### 2.4. Selection Process

Articles retrieved across the search strategy were imported to EndNote X7 (Thomson Reuters). After precluding duplicated articles, titles and abstracts were independently screened by the two review authors (Solomon Getawa and Tiruneh Adane). For articles considered to appear pertinent during title/abstract screening, the full-text was appraised for inclusion in this study. Available discrepancies between the review authors were resolved through consensus, and a third review author (Mulugeta Melku) was involved if required.

### 2.5. Data Extraction

Relevant data from the included studies was summarized into an Excel spreadsheet. The following study characteristics were extracted from the included studies; name of the first author; year of publication; study setting; duration of illness; mean age of the participants; and the NLR value in the good and poor glycemic control groups.

### 2.6. Outcomes of Interest

The main outcome of interest is the comparison of the NLR value between poor and good glycemic control groups (in the form of SMD) in T2DM patients. Six studies divided diabetes control into three groups: group A, with HbA1c 7% (excellent control), group B, with HbA1c 7.0-9.0% (poor control), and group C, with HbA1c 9% (worst control), while the remaining seven studies classified glycemic control into two groups. Those with HbA1c ≤7% (regulated diabetes) were included in group 1, and those with HbA1c >7% (unregulated diabetes) were included in group 2. The secondary outcome is to investigate the association between the NLR values and elevated HbA1c in T2DM patients (in the form of an odd ratio).

### 2.7. Risk of Bias Measurement

A modified Newcastle-Ottawa quality assessment scale was used to evaluate the methodological quality of the included studies [33]. The tool uses 3 sections (selection, comparability, and exposure) to evaluate the quality of case-control studies. Moreover, cohort and cross-sectional studies are also evaluated using 3 sections (selection, comparability, and outcome). Studies with a score of 5 and above are considered high quality.

### 2.8. Statistical Analysis

Results are presented as SMDs with an associated 95% CI. Statistical heterogeneity was measured using the *I*^2^ statistic, with results above 50% considered to be indicative of statistical heterogeneity. A random effect model was employed to estimate the pooled SMD considering the high heterogeneity in the included studies. According to the recommended protocol, studies that reported the NLR value in the form of median and IQR were changed to mean (SD) [34]. Subgroup analysis, metaregression, and sensitivity analysis were conducted to search for potential sources of heterogeneity. The existence of publication bias was assessed qualitatively using funnel plots and quantitatively using the Eggers regression test. A *p* value <0.05 was considered statistically significant. Statistical analyses were performed using STATA 11.0 software.

## 3. Result

### 3.1. Study Selection

**Figure 1:**
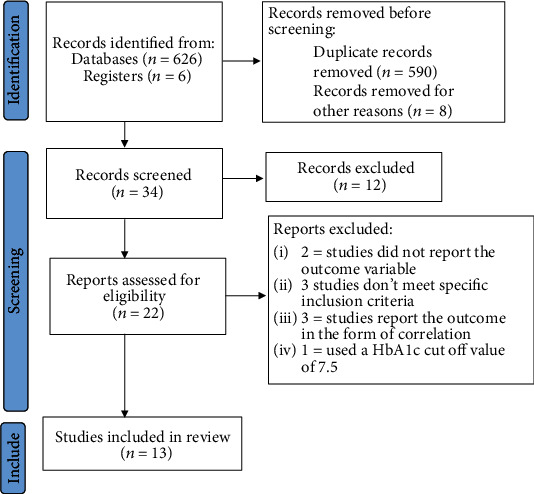
Flow chart of study selection.

A total of 632 abstracts were screened for inclusion. Of the abstracts screened, 598 were excluded as not being relevant and/or duplicates, leaving 34 studies for screening. Finally, 13 studies were included in the qualitative and quantitative analysis (Figure 1).

### 3.2. Study Characteristics

Thirteen studies containing 1,434 participants (623 having good glycemic control and 811 with poor glycemic control) were included. Six studies were conducted in India, 2 in Pakistan, 1 in Italy, 1 in Turkey, 1 in Syria, 1 in Brazil, and 1 in Israel. Five studies followed a cross-sectional study design, one case-control, one retrospective, and four observational studies. Two studies did not report the study design. The results of the Modified Newcastle-Ottawa quality assessment scale showed that very good, good, and satisfactory results were found in 4, 7, and 2 studies, respectively. Their characteristics are summarized in Table 1.

### 3.3. Pooled Mean NLR Value in Poor and Good Glycemic Control T2DM Patients

**Figure 2:**
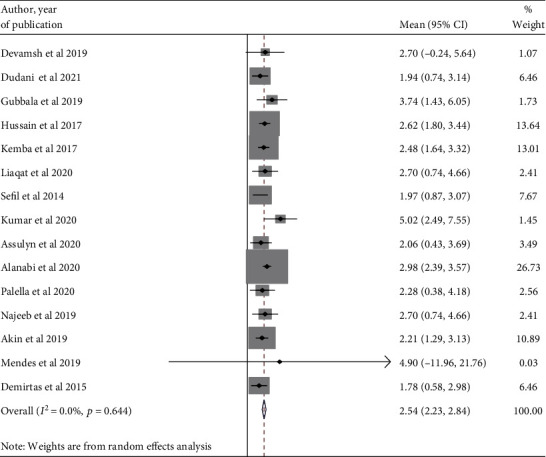
A forest plot displaying the pooled estimate of NLR value among poor glycemic groups.

**Figure 3:**
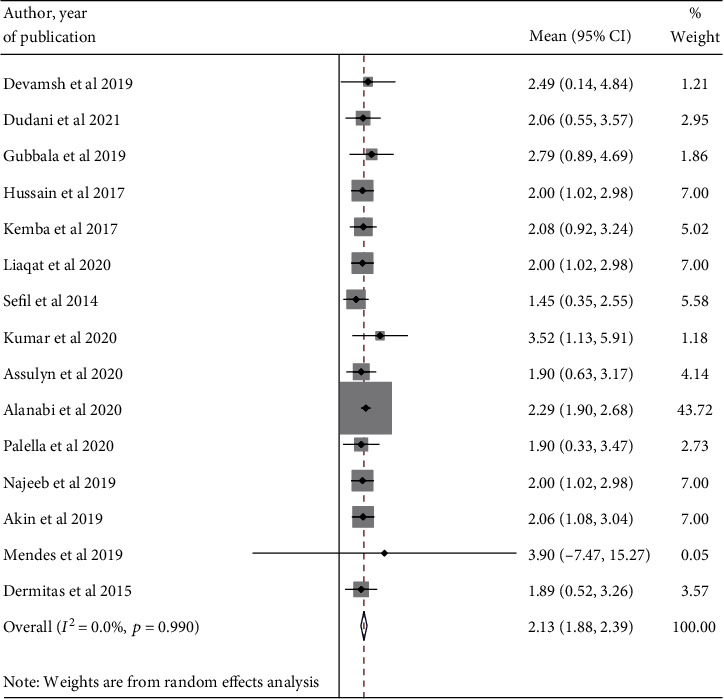
A forest plot displaying the pooled estimate of NLR value among good glycemic groups.

In this study, we tried to determine the pooled mean NLR value in the poor and good glycemic control groups through the random effect model. As a result, the pooled NLR was 2.64 (95% CI: 2.30-2.97) (Figure 2) and 2.15 (95% CI: 1.88-2.42), respectively (Figure 3).

### 3.4. The Association between NLR and Glycemic Control in T2DM Patients

**Figure 4:**
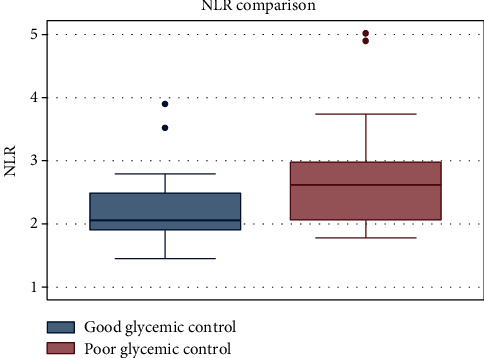
Box plot displaying the comparison of NLR value in the poor and good glycemic control groups.

A total of 13 studies were included in this meta-analysis to explore the association between NLR and glycemic control in T2DM patients. The box plot comparing the NLR value is shown in Figure 4.

**Figure 5:**
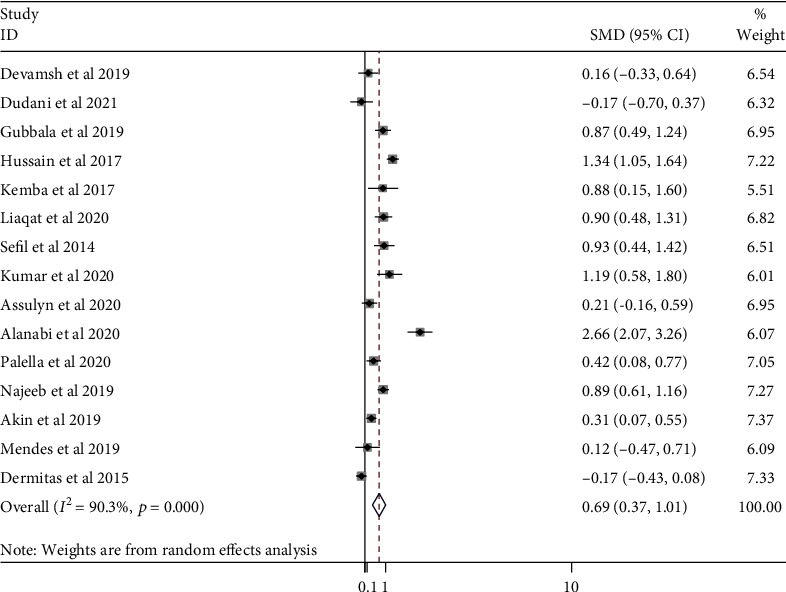
A forest plot showing SMD of the NLR value between good and poor glycemic control in DM patients.

A random effect model was applied because of the significant heterogeneity between studies (*I*^2^ = 86.9%). In the pooled analysis, a significant increase in NLR was observed in the poor control groups than the good control groups (SMD = 0.79; 95% CI, 0.46-1.12; *p* < .001) (Figure 5).

### 3.5. High vs. Low NLR and Glycemic Control in T2DM Patients

**Figure 6:**
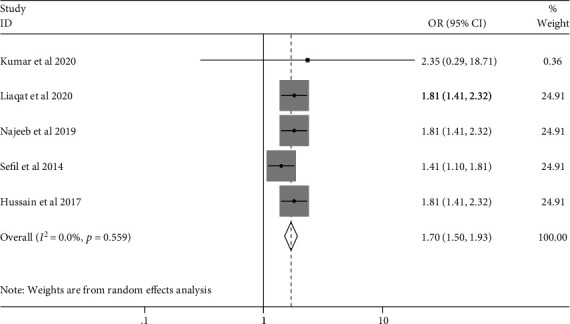
Pooled OR of high NLR value in DM patients.

Five studies reported the odd ratio of a high NLR as an independent predictor of poor and/or worse glycemic control in T2DM patients. The pooled OR was 1.70 (95% CI: 1.50, 1.93) with no heterogeneity (*I*^2^ = 0.0%; *p* value = 0.559) (Figure 6).

### 3.6. Subgroup Analysis

**Figure 7:**
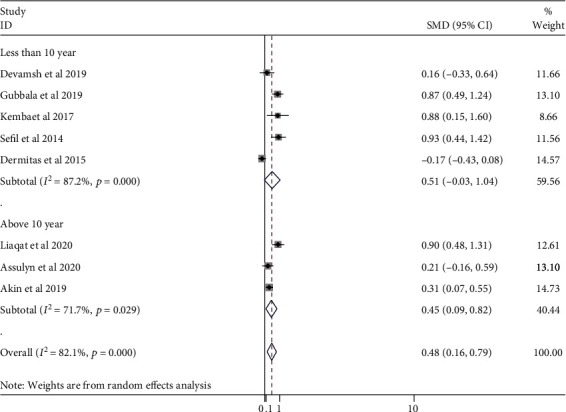
Subgroup analysis stratified by duration of DM.

To explore the sources of heterogeneity, subgroup analysis was carried out according to the duration of illness. Accordingly, the NLR values were 0.70 (95% CI: 0.33, 1.07) and 0.55 (95% CI: -0.12, 1.22) for durations of less than 10 years and above 10 years, respectively (Figure 7).

### 3.7. Sensitivity Analysis

Sensitivity analyses were performed to evaluate the robustness of the results. One study was sequentially omitted at a time to assess its effect on the overall outcome. As a result, no apparent change occurred in the NLR value when an individual study was omitted, confirming that the results were stable (Table 2).

### 3.8. Metaregression

A metaregression was conducted to explore the effect of continuous covariates on differences in the NLR value between poor and good glycemic control groups. The continuous covariates included in the analysis were the year of publication and the duration of illness. Accordingly, any of the covariates show no effect on the pooled SMD of the NLR values (Table 3).

### 3.9. Publication Bias

**Figure 8:**
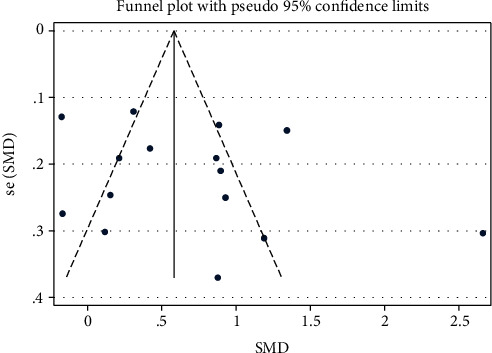
Publication bias.

A funnel plot and Eggers regression tests were performed to explore the presence of publication bias. A visual inspection of the funnel plot shows no divergence from the expected shape (Figure 8); suggesting the absence of publication bias. This is also confirmed by using the Egger tests; *p* value = 0.86 (Table 4).

## 4. Discussion

This systematic review and meta-analysis is aimed at investigating the association between NLR value and glycemic control in T2DM patients. The findings demonstrated that the mean NLR value in the poor group was significantly higher than that of the good glycemic control group (SMD = 0.79; 95% CI, 0.46-1.12; *p* < .001). This study also showed that a high NLR value was significantly associated with poor glycemic control in T2DM patients (OR = 1.50 (95% CI: 1.30-1.93)). This study confirms that the NLR value increased as the HbA1c level worsened and could be a good marker for assessing glycemic control in addition to HbA1c. The increase in NLR in T2DM patients probably showed the inflammatory burden of the disease.

In line with previous studies, this study showed that the NLR value could be used as a marker of diabetic control level besides the HbA1c level in T2DM patients [47, 48]. It can be associated with the negative effects of neutrophils on endothelial damage and the antiatherosclerotic role of lymphocytes [49]. Chronic inflammation in T2DM progresses with leukocyte recruitment to the vascular environment in response to oxidative stress and the production of proinflammatory cytokines [50]. The power of the NLR value as an inflammatory factor stems from both a reduction in the lymphocyte count and an increase in the neutrophil count [51]. Neutrophils rapidly respond to inflammatory stimuli and increase their number in circulation. Studies showed that there is an increased expression of activation markers like CD11b/CD18 on monocytes and neutrophils in T2DM patients, resulting in increased neutrophil adhesiveness to the endothelium, independent of fasting glucose levels [52, 53]. Leucocytes in DM patients may also be activated by leptin and advanced glycation end products [54]. Activated leucocytes then contribute to systemic inflammation and endothelial damage by releasing reactive oxygen species through neutrophils and cytokines [55]. In addition, the relative number of regulatory T cells compared to helper T cells is reduced in patients with DM [56]. Increased interleukin levels during inflammation cause lymphopenia [23] and neutrophilia [24], together resulting in a high NLR value. It is associated with microvascular and macrovascular complications of DM and metabolic impairment [57].

The current study showed that the NLR is significantly related to the level of hyperglycemia in T2DM patients. Previous research has linked high NLR levels to elevated HbA1c levels in T2DM [31, 41]. Clinicians measure the long-term glycemic control in DM patients using the HbA1c test. However, HbA1c may be affected by a variety of genetic, hematologic, and illness-related factors. Hemoglobinopathies, certain anemia, and disorders associated with accelerated red cell turnover, such as malaria, are the most common important factors affecting HbA1c levels worldwide [58]. Furthermore, recent blood transfusion, use of erythropoiesis-stimulating drugs, end-stage kidney disease, and pregnancy may cause discrepancies between the HbA1C result and the patient's true mean glycaemia [59]. The NLR has been identified as a potential marker to determine inflammation in various cardiac and noncardiac disorders because it has a superior predictive, diagnostic, and discriminative ability than the total WBC count [36]. It is a simple and inexpensive test for assessing inflammation that is obtained by dividing the absolute neutrophil to absolute lymphocyte count [60].

Aside from diabetes, the NLR value is used to predict the prognosis of other inflammatory diseases, including cardiovascular disease [61], gestational diabetes mellitus [62], chronic obstructive pulmonary disease [63], hypertension [64], and colorectal cancer [65]. It has also proven its usefulness in the stratification of mortality in major cardiac events, as a strong prognostic factor in several types of cancer, or as a predictor and marker of inflammatory or infectious pathologies (such as pediatric appendicitis) and postoperative complications [66]. Increased NLR values and the risk of cardiovascular events are explained by neutrophils secreting inflammatory mediators that can cause vascular wall degeneration [67] and lymphocytes regulating the inflammatory response and acting as antiatherosclerotic agents [68]. Increased neutrophil and decreased lymphocyte count in hypertensive complications such as neuropathy, cardiomyopathy, and retinopathy occurred due to inflammatory response developed in the arterial walls due to elevated pressures [69, 70]. Recent evidence has shown that a high NLR value can be used to predict inhospital and postdischarge mortality in chronic obstructive pulmonary disease patients [63]. Elevated NLR and poor prognosis have been reported in different cancer patients due to inflammation-associated elevation of tumor-associated neutrophils or neutrophils which infiltrate tumors [71, 72].

Subgroup analysis of this study revealed that the duration of diabetes had no statistically significant difference in NLR value to predict glycemic control in diabetic patients. The findings were consistent with previous research [38, 41]. Though the pooled estimate did not show a significant difference in NLR between the poor and good glycemic control groups, studies by Chittawar et al. and Gubbala et al. found that the duration of T2DM and NLR were significantly involved in determining the glycemic control of DM patients [12, 73]. This is because T2DM patients are more likely to develop microvascular complications, which result in higher blood pressure, NLR, creatinine, and albumin levels as the illness progresses [73].

The current study has some strengths and limitations. The strength of the study is its comprehensive literature search by the two independent authors to extract all available published articles. To the best of our knowledge, this is the first systematic review and meta-analysis to address the association between NLR and glycemic control in T2DM patients. Even though we did metaregression, subgroup, and sensitivity analysis, the heterogeneity was high. This might be due to the inclusion of studies only in the English language. Besides, the study cannot address the prognostic and diagnostic role of NLR in the glycemic control of T2DM patients.

## 5. Conclusions and Recommendations

The results of this study showed that there was a higher NLR value in poor glycemic control patients than in their counterparts. This suggests an association of high NLR values with an elevated HbA1c in T2DM patients. Therefore, NLR should be considered a marker of glycemic control in addition to HbA1C in T2DM patients.

## Figures and Tables

**Table 1:** Descriptive summary of the included studies on the role of NLR as glycemic control in T2DM patients.

Author, publication year	Country	Sample size		NLR		Duration of illness		Quality score
		Good control	Poor control	Good control	Poor control	Good control	Poor control	
Dudani et al., 2021 [35]	India	40	20	2.06 ± 0.77	1.94 ± 0.61	—	—	Good
Devamsh and Raghavan, 2019 [36]	India	33	33	2.49 ± 1.2	2.701 ± 1.5	6.93 ± 5.3	6.84 ± 3.6	Satisfactory
Mendes et al., 2019 [37]	Brazil	12	127	3.9 ± 5.8	4.9 ± 8.6			Good
Gubbala et al., 2019 [12]	India	53	69	2.79 ± 0.97	3.74 ± 1.18	6.2 ± 4.2	8.9 ± 6.8	Good
Hussain et al., 2017 [38]	Pakistan	110	110	2.0 ± 0.5	2.62 ± 0.42	—	—	Very good
Kemba, 2017 [39]	India	9	51	2.08 ± 0.59	2.48 ± 0.43	5.55 ± 3.33	5.94 ± 3.17	Good
Liaqat et al., 2020 [40]	Pakistan	52	48	2 ± 0.5	2.7 ± 1.0	14 ± 20	17 ± 24	Satisfactory
Sefil et al., 2014 [41]	Turkey	34	37	1.45 ± 0.56	1.97 ± 0.56	7 ± 6.3	6.5 ± 5.9	Very good
Kumar et al., 2020 [42]	India	21	29	3.52 ± 1.22	5.02 ± 1.29	—	—	Good
Assulyn et al., 2020 [43]	Israel	53	57	1.90 ± 0.65	2.06 ± 0.83	10 ± 6	14 ± 8	Very good
Alnabi and Hussain, 2020 [44]	Syria	38	45	2.29 ± 0.2	2.98 ± 0.3	—	—	Good
Palella et al., 2020 [45]	Italy	58	75	1.90 ± 0.8	2.28 ± 0.97	—	—	Good
Najeeb, 2019 [46]	India	110	110	2.0 ± 0.5	2.7 ± 1.0	—	—	Very good

**Table 2:** Sensitivity analysis.

Excluded studies	SMD (95% CI)	Heterogeneity	
		*I* ^2^	*p* value
Dudani et al., 2021 [35]	0.87 (0.54-1.19)	86%	≤0.001
Devamsh and Raghavan, 2019 [36]	0.84 (0.50-1.19)	87%	≤0.001
Mendes et al., 2019 [37]	0.84 (0.50-1.18)	87.3%	≤0.001
Gubbala et al., 2019 [12]	0.79 (0.42-1.15)	88%	≤0.001
Hussain et al., 2017 [38]	0.74 (0.40-1.08)	85.5%	≤0.001
Kemba, 2017 [39]	0.79 (0.44-1.13)	88%	≤0.001
Liaqat et al., 2020 [40]	0.78 (0.43-1.14)	88%	≤0.001
Sefil et al., 2014 [41]	0.78 (0.43-1.13)	88%	≤0.001
Kumar et al., 2020 [42]	0.76 (0.42-1.11)	87.8%	≤0.001
Assulyn et al., 2020 [43]	0.84 (0.50-1.19)	86.5%	≤0.001
Alnabi and Hussain, 2020 [44]	0.66 (0.39-0.92)	79.1%	≤0.001
Palella et al., 2020 [45]	0.83 (0.47-1.18)	87.3%	≤0.001
Najeeb, [46]	0.67 (0.32-1.02)	90.7%	≤0.001
Combined	0.79 (0.46-1.12)	86.9%	≤0.001

**Table 3:** Metaregression.

Variables	Coefficient (95% CI)	*p* value
Year of publication	0.016 (-0.197,0.23)	0.870
Duration of illness (poor glycemic control)	0.003 (-0.09, 0.092)	0.933
Duration of illness (good glycemic control)	0.002 (-0.12, 0.13)	0.965

**Table 4:** Egger's test.

	Standard effect	Coefficient	Standard error	*p* > |*t*|	(95% confidence interval)
Slope	0.90	0.60	1.50	0.16	-0.42, 2.23
Bias	-0.51	2.8	-0.18	0.86	-6.77, 5.74

## Data Availability

All data generated or analyzed during this study are included in this published article.

## Abbreviations

DM: Diabetes mellitus
HbA1c: Plasma glycated hemoglobin
IL-6: Interleukin-6
NLR: Neutrophil-to-lymphocyte ratio
SMD: Standardized mean difference
T2DM: Type 2 diabetes mellitus.
